# Advanced Endoscopic Management of a Full-Thickness Esophageal Perforation From Food Impaction: A Case of Successful Stent-Assisted Healing

**DOI:** 10.7759/cureus.87021

**Published:** 2025-06-30

**Authors:** Usman Bin Hameed, Mujtaba Moazzam, Joel Karsten, Fady Banno, Aleena Sharif

**Affiliations:** 1 Internal Medicine, Corewell Health William Beaumont University Hospital, Royal Oak, USA; 2 Internal Medicine, Oakland University William Beaumont School of Medicine, Auburn Hills, USA; 3 Gastroenterology, Corewell Health William Beaumont Hospital, Royal Oak, USA; 4 Internal Medicine, Sheikh Zayed Medical College, Rahim Yar Khan, PAK

**Keywords:** argon plasma photocoagulation, endoscopic clip, endoscopic vacuum therapy, fully covered self-expanding metal stents, spontaneous esophageal perforation

## Abstract

Esophageal perforation is a rare but life-threatening condition with historically high morbidity and mortality. Traditional management has relied on urgent surgical repair, but advances in endoscopic therapy have expanded non-surgical options. We present a case of an 84-year-old male patient with an esophageal perforation caused by food impaction in the setting of a Schatzki ring, successfully managed through a stepwise endoscopic approach using a fully covered self-expanding metal stent (FCSEMS) followed by argon plasma coagulation (APC) and endoscopic clipping to close a residual defect.

The patient with risk factors including advanced age, chronic esophageal strictures, and a history of repeated dilations presented with acute dysphagia and chest pain following food impaction. Imaging revealed a distal esophageal perforation with pneumomediastinum and developing mediastinal fluid collection. Esophagogastroduodenoscopy (EGD) identified an impacted food bolus and a 1 cm perforation surrounded by necrotic debris. Endoscopic debridement was performed, and a fully covered 23 mm × 120 mm EndoMAXX esophageal stent (Merit Medical Systems, Inc., South Jordan, UT) was deployed under fluoroscopic guidance to seal the defect.

At the two-week follow-up EGD, the stent remained in position with granulation tissue formation. Due to persistent dysphagia, likely secondary to stent-related granulation tissue, mucosal inflammation, or mechanical irritation, the stent was removed. At eight weeks, a small residual 3 mm perforation was visualized. This was treated with APC (1.2 L/min, 20 W) to ablate epithelialized edges, followed by mechanical closure using two MANTIS clips (Boston Scientific Corporation, Marlborough, MA). A percutaneous endoscopic gastrostomy-jejunostomy (PEG-J) tube was surgically placed for enteral support. A 10-week endoscopy confirmed mucosal healing with embedded clips and no surrounding inflammation. At 16 weeks, an esophagram demonstrated complete closure without a leak. The PEG-J tube was removed, and the patient resumed normal oral intake, underscoring the effectiveness of stepwise endoscopic management for esophageal perforation.

This case illustrates the effective use of multimodal endoscopic therapy for esophageal perforation. Fully covered stent placement achieved initial closure and containment, while a secondary endoscopic intervention (APC with clipping) successfully sealed a persistent micro-perforation. The sequential minimally invasive approach obviated the need for thoracic surgery and its associated risks. Advances in endoscopic stents, tissue ablation, and clipping devices offer a paradigm shift in managing esophageal perforations, especially in elderly or high-risk patients.

## Introduction

Esophageal perforation is a rare but life-threatening emergency, occurring in approximately one per 100,000 people annually in the United States, with the majority of cases now being iatrogenic (~60%) due to endoscopic or surgical interventions [1]. Prompt diagnosis is critical, as mortality sharply increases with delays beyond 24 hours from around 10% to 25% when intervention is early to approximately 40% to 60% if delayed [2,3]. Historically, surgical management, including primary repair, drainage, esophagectomy, or exclusion procedures, formed the cornerstone of treatment; however, these methods carry substantial morbidity and notable mortality, ranging from approximately 10% to 25%, with significant postoperative complications such as pulmonary infections and sepsis in roughly 10% to 15% of cases [4]. Advances in endoscopic therapies, including fully covered self-expanding metal stents (FCSEMS), over-the-scope clips (OTSC), endoscopic suturing, and endoluminal vacuum-assisted therapy (EVT), have significantly shifted the therapeutic paradigm. FCSEMS placement now achieves successful leak closure rates of approximately 80% to 90% and carries significantly lower mortality rates of about 6% to 16%, though complications like stent migration occur in up to 20% of cases [5]. EVT, despite promising results in carefully selected cases, often necessitates multiple sessions and careful patient selection. Current practice increasingly favors individualized treatment approaches, utilizing less invasive endoscopic techniques alongside surgery, resulting in improved survival and reduced morbidity. Mortality rates with contemporary multidisciplinary management have declined to approximately 10% to 15%, reflecting significant progress in managing this critical condition [1,5,6].

## Case presentation

An 84-year-old male patient with a history of hiatal hernia, gastroesophageal reflux disease (GERD), esophageal strictures, and a known Schatzki ring, status post multiple dilations (most recently in 2022), presented to the emergency department with acute dysphagia and severe retrosternal chest pain immediately after eating chicken wings. He experienced an inability to swallow saliva, significant diaphoresis, nausea, and an acute onset of chest discomfort radiating to the back. On initial examination, he was alert but appeared moderately distressed. Vital signs included blood pressure 151/58 mmHg, pulse 63 beats per minute, temperature 97.6°F (axillary), and respiratory rate 16 breaths per minute. Physical examination revealed palpable crepitus in the supraclavicular region. Initial laboratory evaluation (Tables 1-2) showed leukocytosis (white blood cell (WBC) count: 20.9 bil/L), anemia (hemoglobin: 12 g/dL, hematocrit: 36.7%), hyponatremia (sodium: 133 mmol/L), metabolic acidosis (bicarbonate: 16 mmol/L), significantly elevated amylase (713 U/L), elevated glucose (153 mg/dL), and hypocalcemia (calcium 7.9 mg/dL). Liver function tests were normal except for mildly low alanine aminotransferase (ALT) at 7 U/L. Urinalysis indicated turbid urine with 2+ blood, protein 30 mg/dL, and >20 RBC/hpf. ECG and troponins were negative, and infectious workup showed no evidence of infection. 

**Table 1:** Complete blood count report Demonstrating significant leukocytosis and mild normocytic anemia

Laboratory results	Admission	Reference range and units
White blood bells	20.9	3.5 - 10.1 bil/L
Red blood cells	4.02	4.31 - 5.48 tril/L
Hemoglobin	12	13.5 - 17 g/dL
Hematocrit	36.7	40.1 - 50.1%
Mean corpuscular volume (MCV)	91	80 - 100 fL
Mean corpuscular hemoglobin concentration (MCHC)	33	32 - 36 g/dL
Red blood cell distribution width (RDW)	14	12 - 15%
Platelets	259	150 - 400 bil/L

**Table 2:** Basic metabolic profile The reports demonstrated mild hyponatremia, elevated serum amylase, and normal renal function.

Laboratory results	Admission	Reference range and units
Sodium	133	135 - 145 mmol/L
Potassium	4.2	3.5 - 5.2 mmol/L
Chloride	107	98 - 111 mmol/L
Bicarbonate	16	20 - 29 mmol/L
Anion gap	10	5 – 17
Glucose	153	60 - 99 mg/dL
Blood urea nitrogen	19	7 - 25 mg/dL
Creatinine	0.96	0.50 - 1.10 mg/dL
Estimated glomerular filtration rate (eGFR)	78	> 90 mL/min/1.73m^2^
Calcium	7.9	8.5 - 10.5 mg/dL
Protein total	6.3	6.4 - 8.3 g/dL
Uric acid	5.6	3.5 - 7.2 mg/dL
Amylase	713	<100 U/L

**Figure 1:**
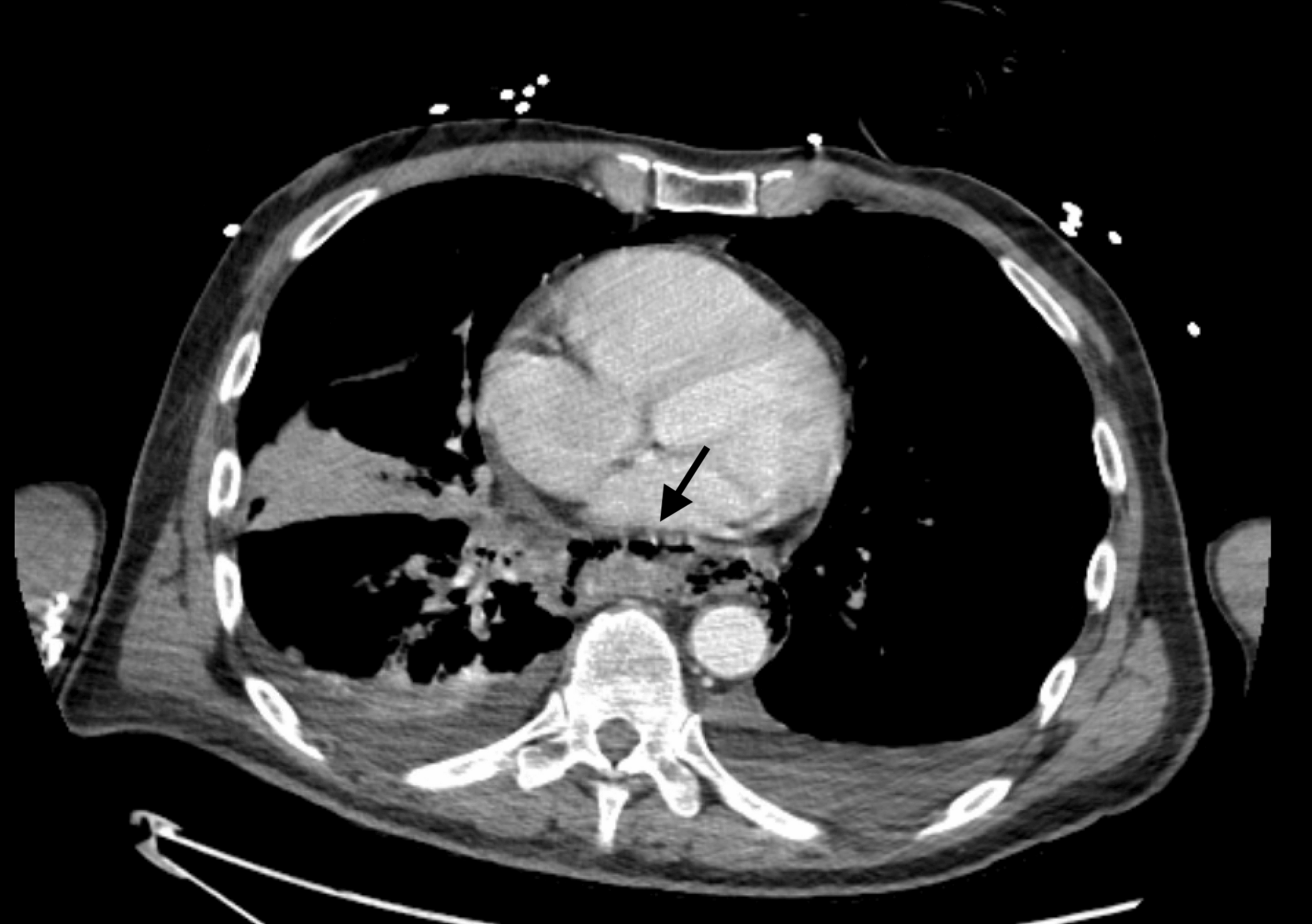
Axial CT of the chest with intravenous contrast demonstrated pneumomediastinum with extraluminal air tracking along the distal esophagus and mediastinal fat stranding, consistent with esophageal perforation. A gas-containing collection was seen adjacent to the esophagus, suggestive of a walled-off leak or developing abscess.

Initial imaging, including chest CT, demonstrated distal esophageal perforation, significant pneumomediastinum, pneumoperitoneum, and mediastinal inflammation without significant abscess formation (Figure 1). Additionally, there was at least 5.0 cm of gas and fluid collection intimately associated with the distal left esophagus, suggestive of a walled-off portion of the perforation or developing abscess, favoring an esophageal tear near the level of the diaphragm.

**Figure 2:**
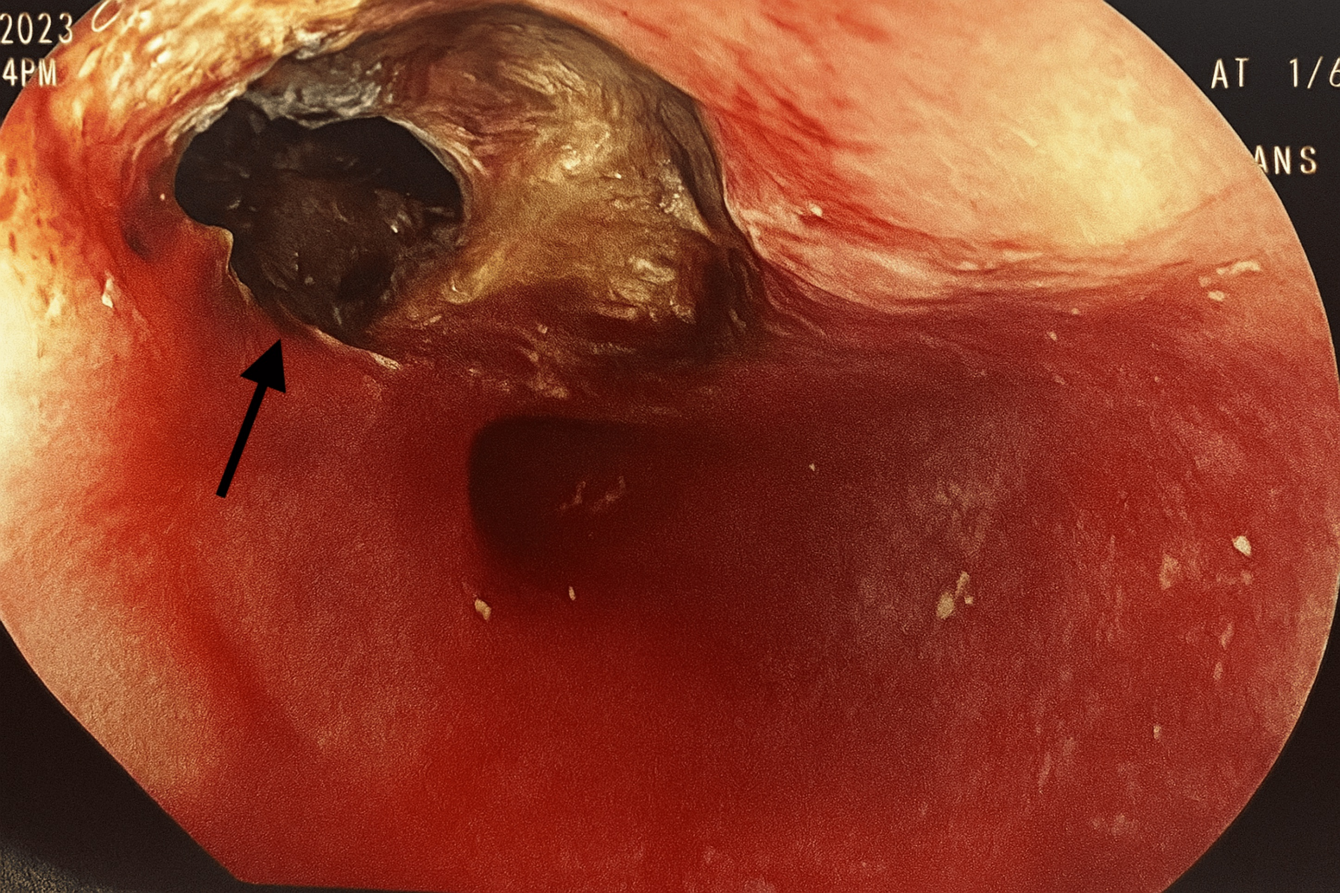
Esophagogastroduodenoscopy (EGD) of the distal esophagus revealed a large ulceration with overlying necrotic debris.

Subsequent urgent endoscopy revealed a distal esophageal ulceration measuring approximately 1 cm in diameter, surrounded by necrotic mediastinal debris secondary to an impacted food bolus (Figure 2).

**Figure 3:**
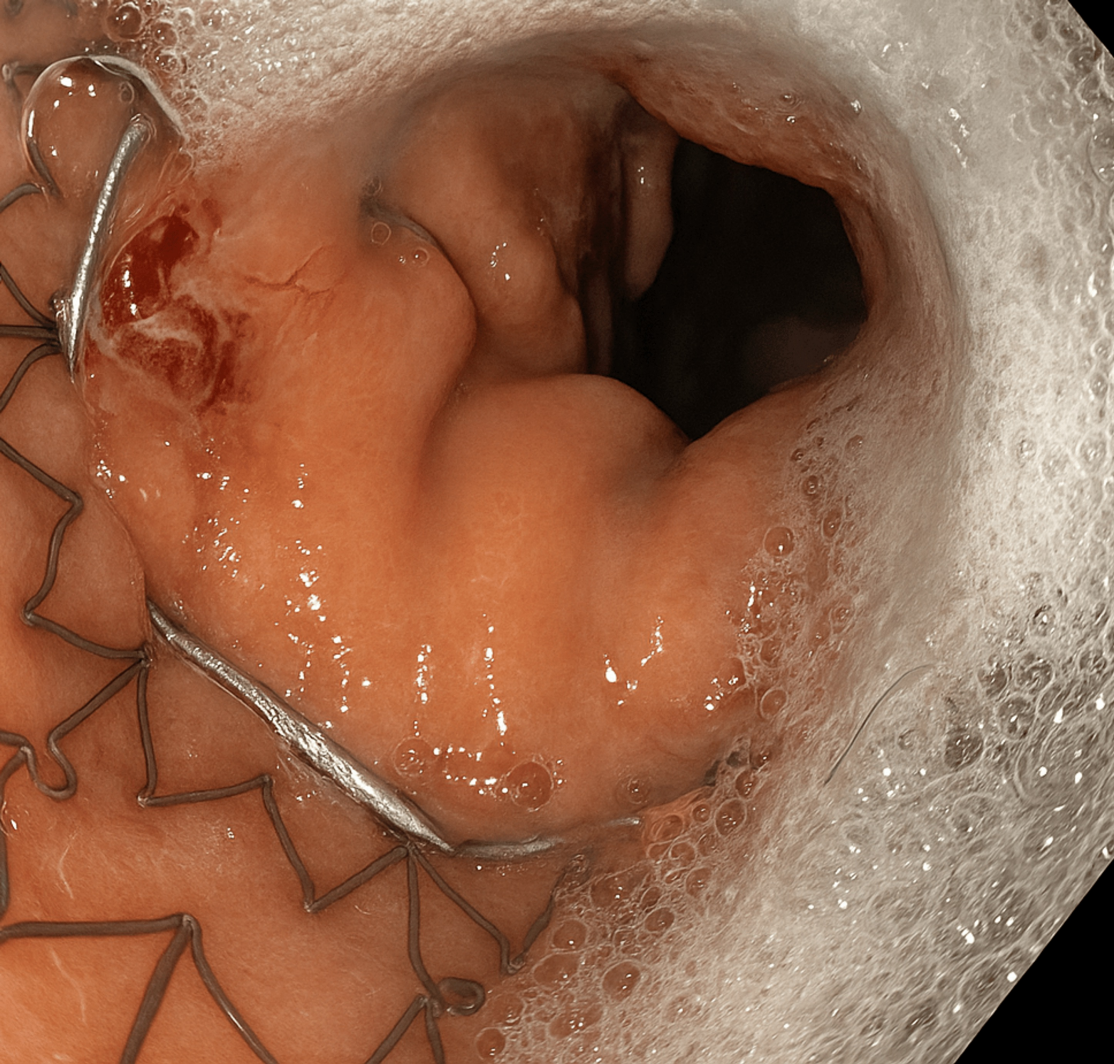
Endoscopic view of the distal esophagus A fully covered esophageal stent was placed, bridging the area of perforation.

During this endoscopy, the impacted chicken meat was removed, and necrotic mediastinal debris was cleared. A fully covered 23 mm × 120 mm Merit EndoMAXX esophageal stent (Merit Medical Systems, Inc., South Jordan, UT) was placed endoscopically across the defect under fluoroscopic and direct visualization, effectively sealing the perforation (Figure 3). No additional anchoring methods beyond the inherent anti-migration design features of the stent were utilized, as this was the initial stent placement with no prior migration concerns.

**Figure 4:**
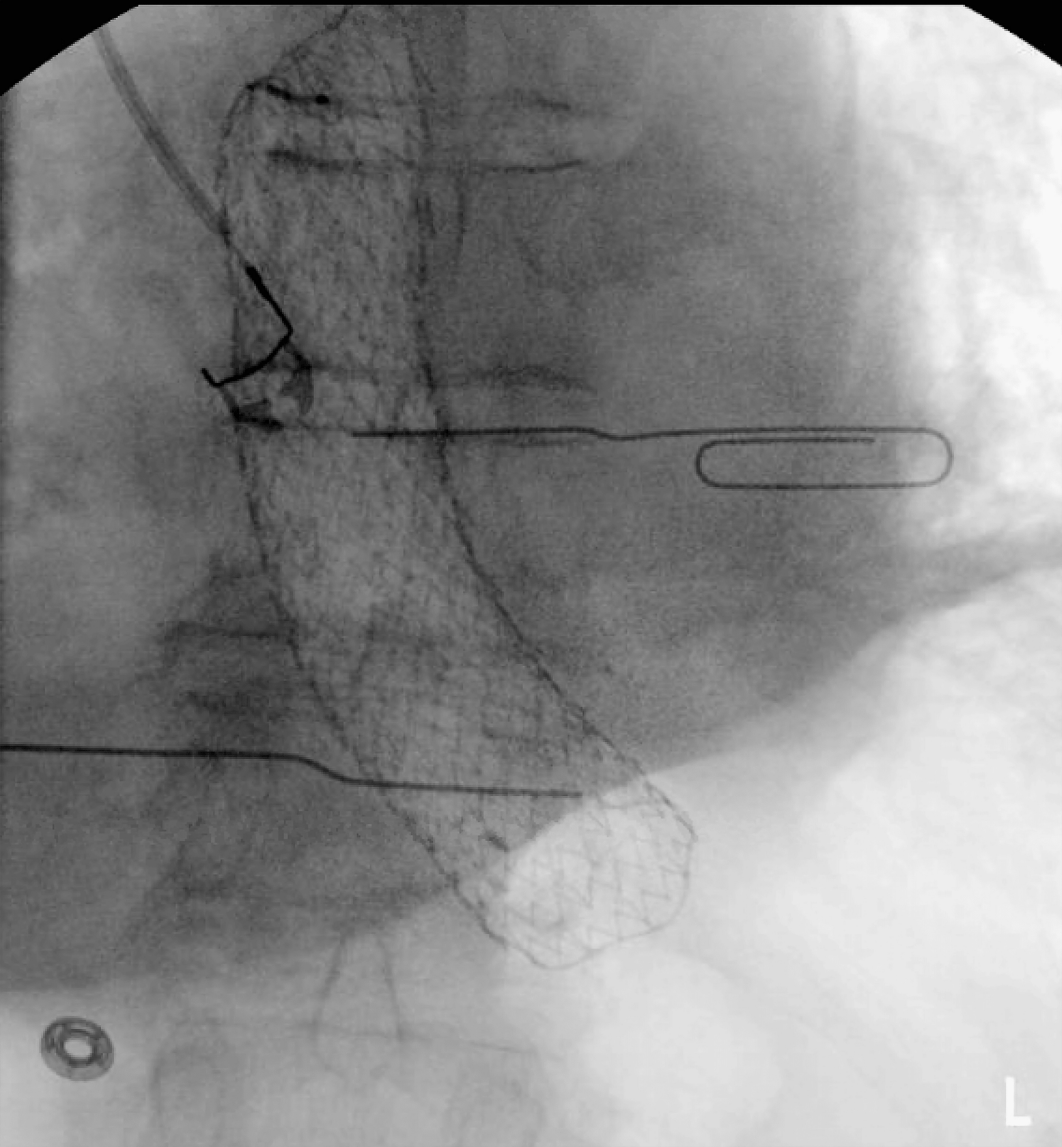
Fluoroscopic image of the distal esophagus demonstrated a fully covered esophageal stent in an appropriate position bridging the site of perforation.

**Figure 5:**
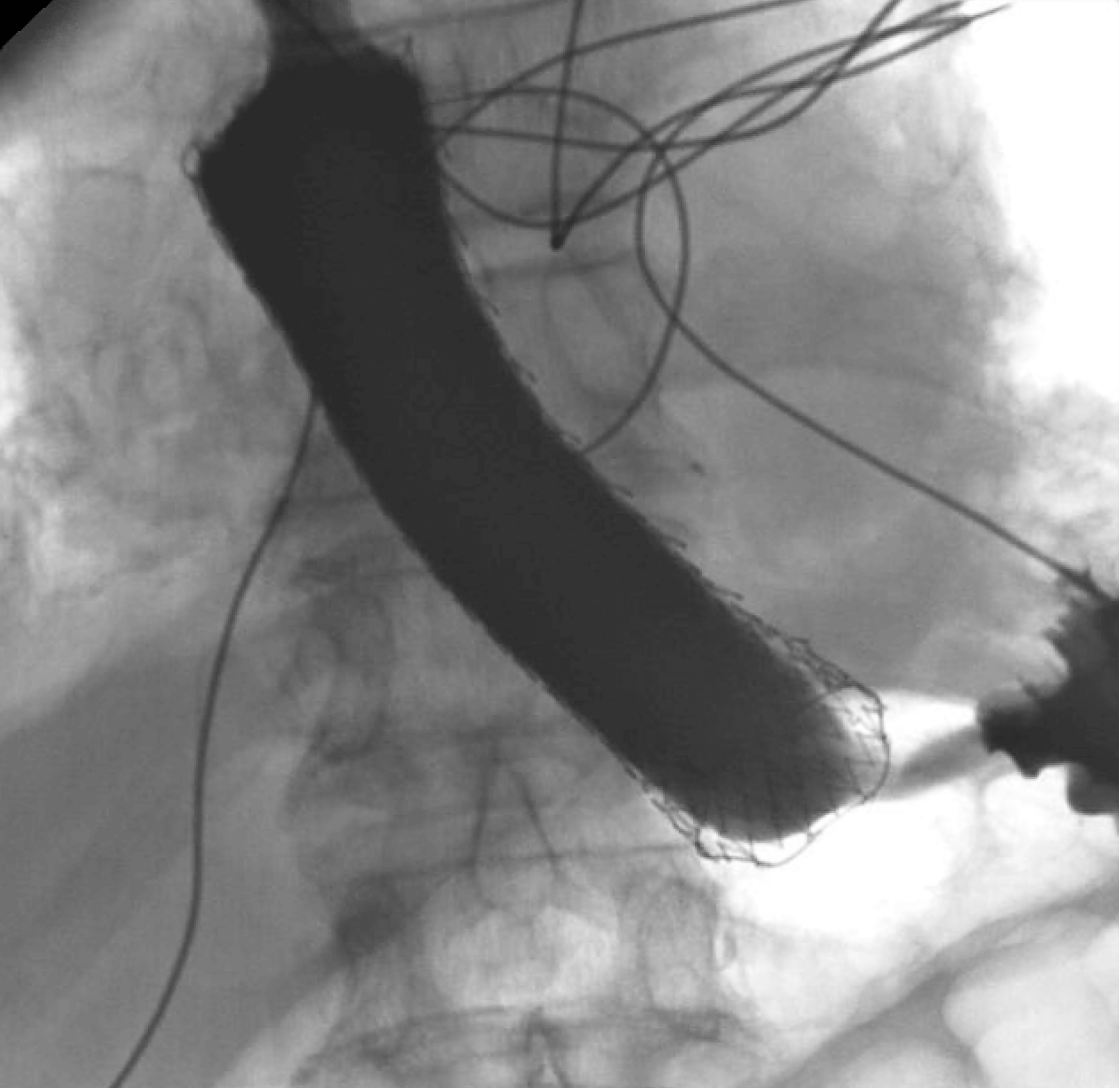
Contrast fluoroscopic image of the esophagus with contrast injection showed a fully covered esophageal stent in situ with no evidence of extravasation, confirming effective sealing of the esophageal perforation.

The stent's proper placement was confirmed by injection of contrast demonstrating no extravasation along with fluoroscopic confirmation (Figures 4, 5).

The patient was subsequently transferred for further management, requiring intubation due to hypotension, and admitted to the medical intensive care unit (MICU) with initiation of vasopressors.

**Figure 6:**
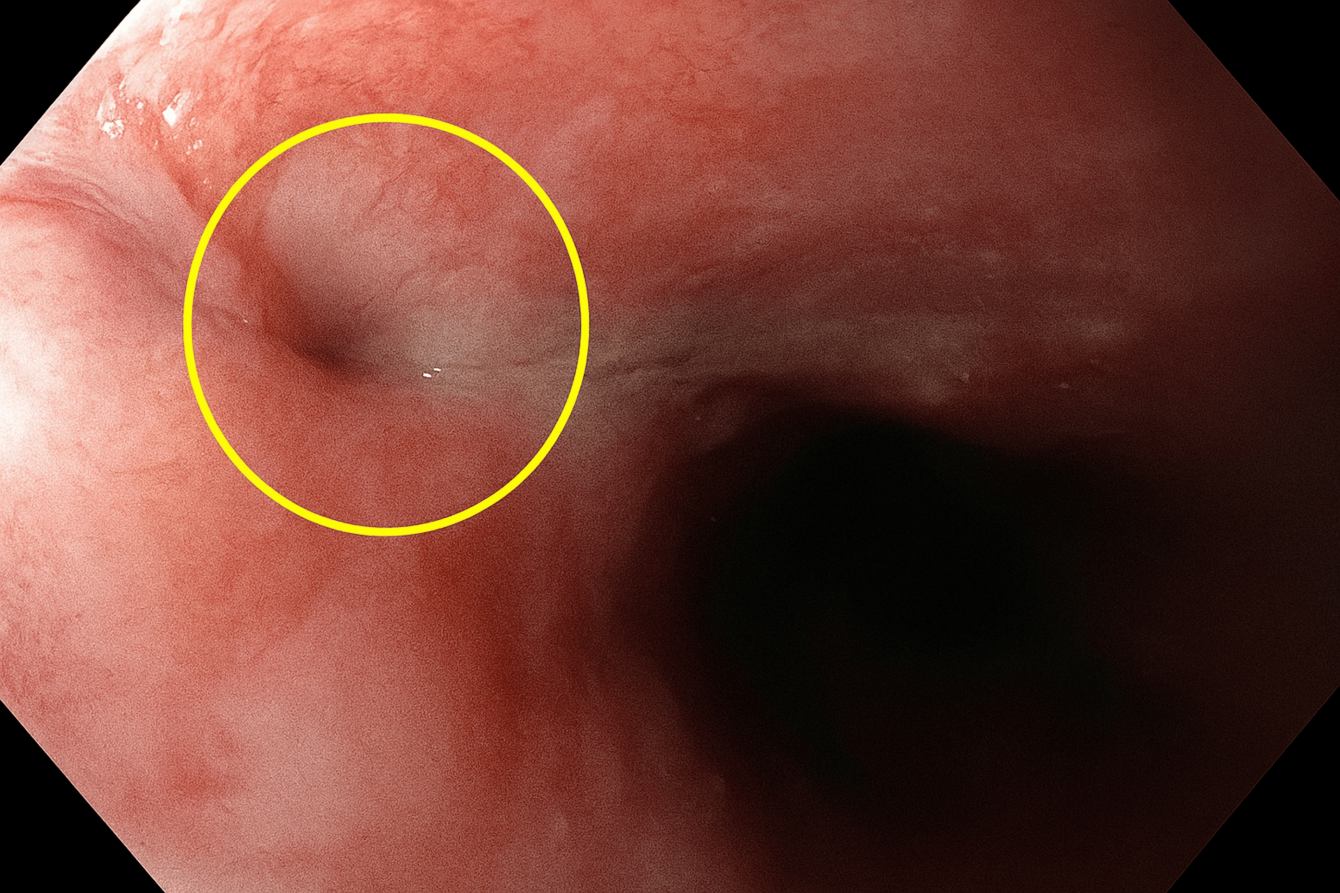
Endoscopic view of the distal esophagus demonstrated a small residual perforation (circled) measuring approximately 3 mm in diameter, surrounded by mildly inflamed mucosa and early granulation tissue.

At two weeks, follow-up esophagogastroduodenoscopy (EGD) confirmed optimal stent position and significant granulation tissue formation indicative of healing; however, the patient reported persistent dysphagia and discomfort, necessitating stent removal. At eight weeks, a repeat EGD revealed a small residual perforation of approximately 3 mm in diameter, surrounded by mild inflammation and granulation tissue (Figure 6).

**Figure 7:**
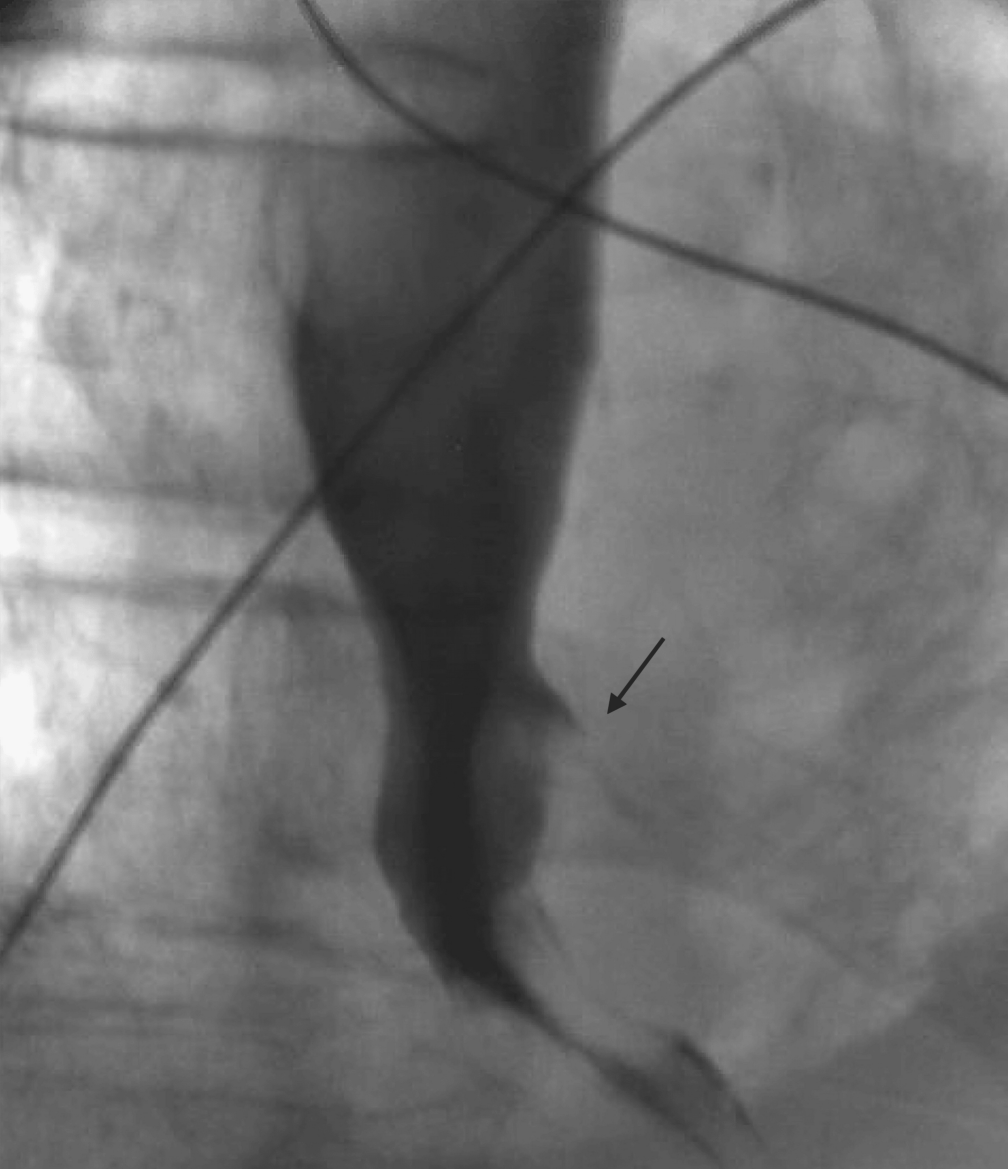
Fluoroscopic esophagram demonstrated a small contrast leak (arrow) at the distal esophagus, consistent with a residual esophageal perforation following stent removal.

Fluoroscopy confirmed a minimal leak at the distal esophageal wall (Figure 7 and Video 1).

**Video 1:** Fluoroscopic esophagram showed dynamic visualization of contrast extravasation at the distal esophagus, consistent with a residual perforation following stent removal. The leak was subtle and visualized during contrast passage into the stomach.

**Figure 8:**
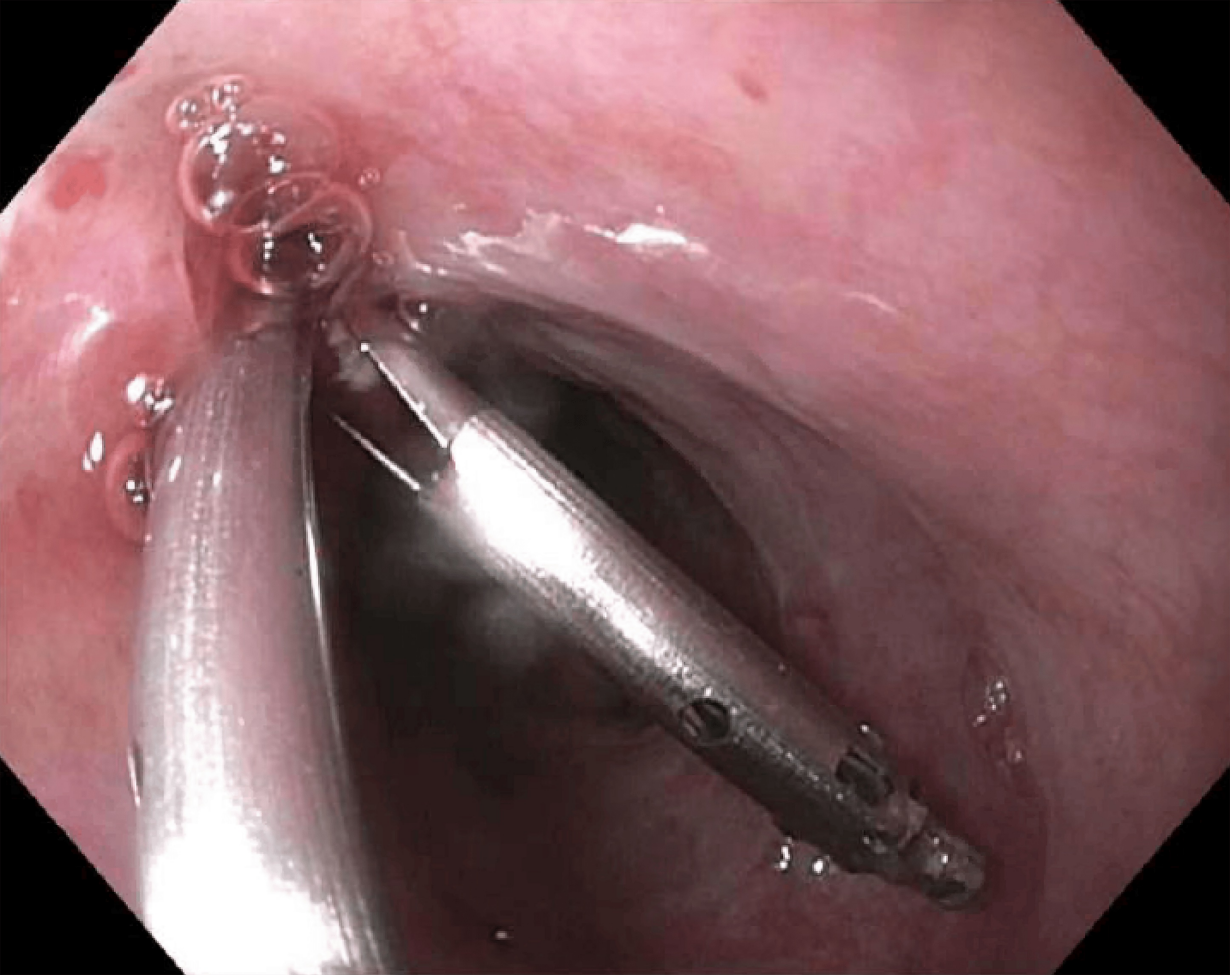
Endoscopic image of the distal esophagus during closure Deployment of a MANTIS endoscopic clip across the residual esophageal defect to approximate tissue edges and promote healing following argon plasma coagulation (APC).

The area surrounding the residual perforation was treated with APC using settings of 1.2 L/min at 20 watts to ablate mucosal margins, followed by mechanical closure using two MANTIS endoscopic clips (Boston Scientific Corporation, Marlborough, MA) carefully deployed to approximate and reinforce the tissue edges (Figure 8).

**Figure 9:**
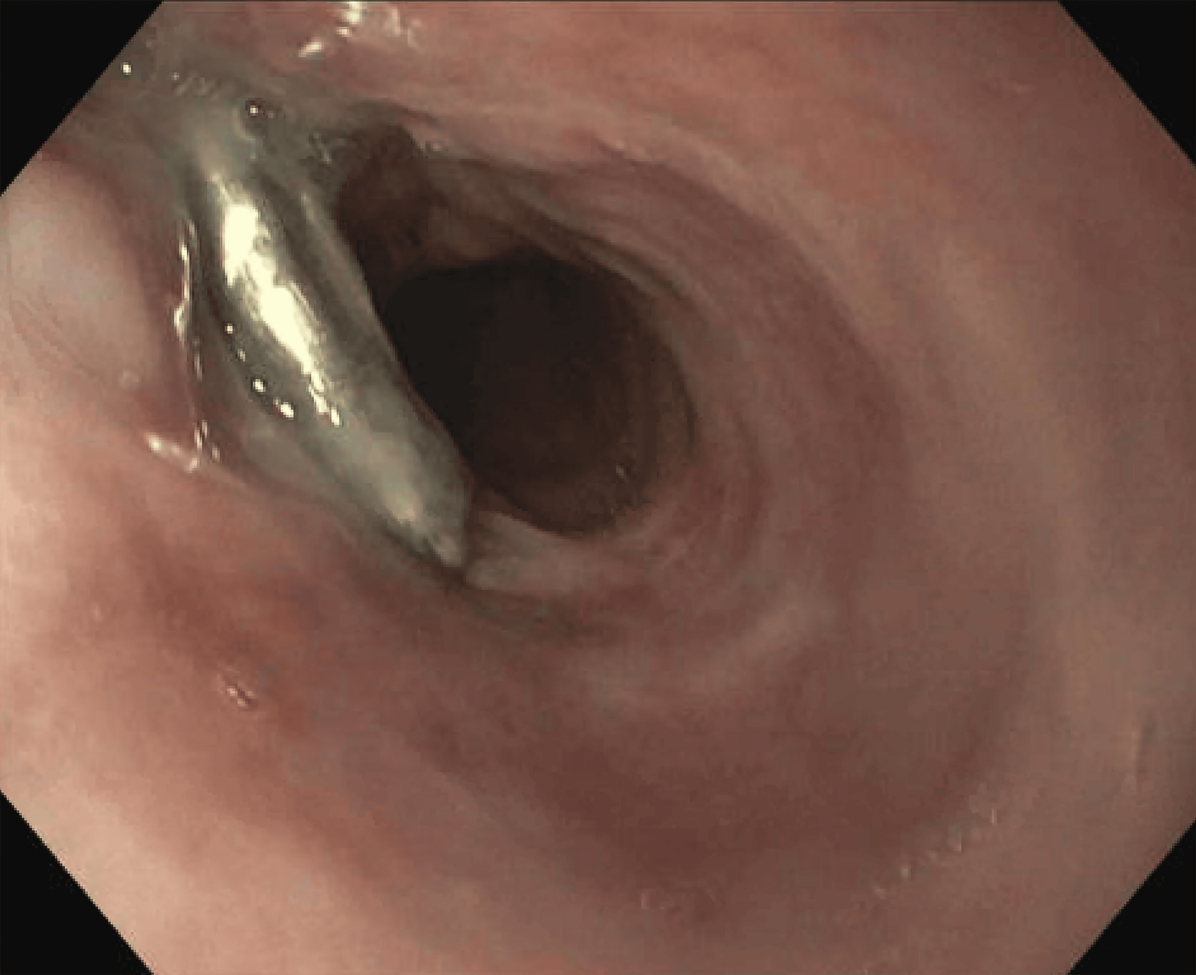
Endoscopic view of the distal esophagus at 10-week follow-up showed healing of the mucosal defect with a partially embedded MANTIS clip in place; the surrounding mucosa appeared healthy with no evidence of active inflammation, granulation tissue, or stricture.

Subsequently, a percutaneous endoscopic gastrostomy-jejunostomy (PEG-J) tube was placed surgically for enteral nutrition to facilitate healing. At 10 weeks, follow-up endoscopy confirmed a healed mucosal defect with a partially embedded MANTIS clip. Surrounding mucosa appeared healthy, with no signs of inflammation, stricture, or granulation tissue. Findings were consistent with appropriate interval healing (Figure 9).

**Figure 10:**
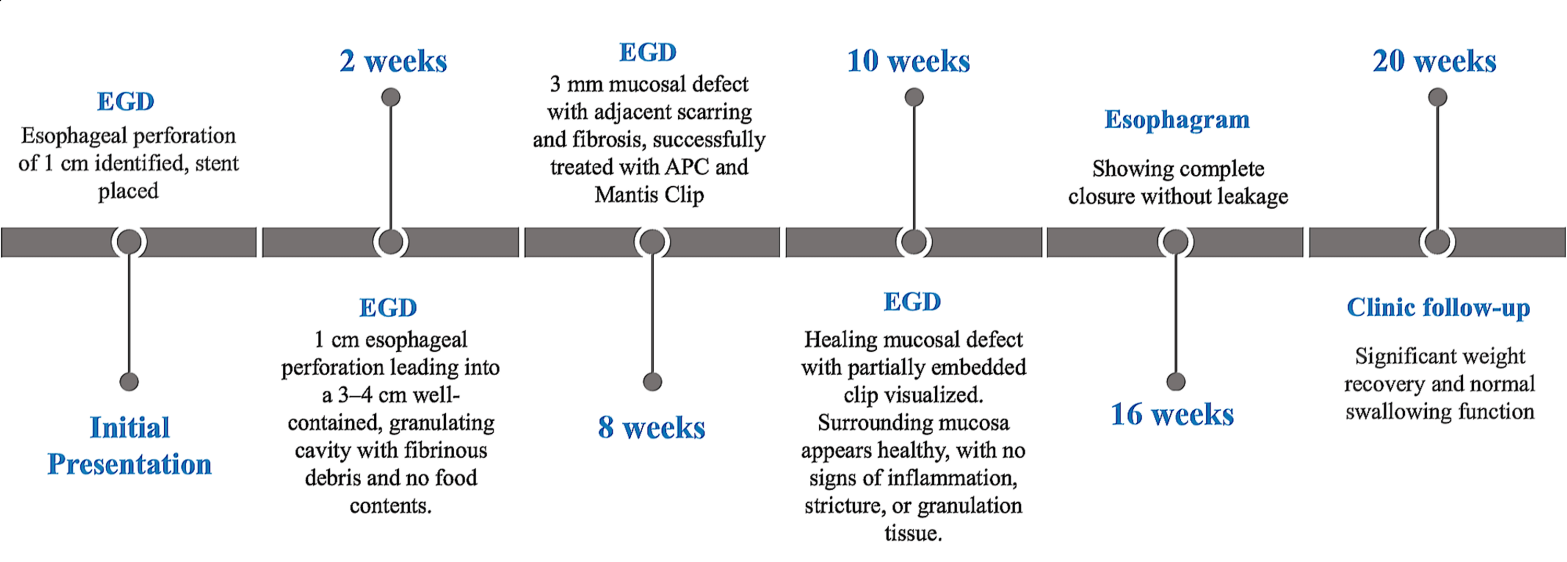
Clinical timeline of endoscopic management of the esophageal perforation Stepwise progression from initial stent placement to complete mucosal healing over 20 weeks. Interventions included serial esophagogastroduodenoscopy (EGD), argon plasma coagulation (APC) treatment, MANTIS clip placement, and A percutaneous endoscopic gastrostomy-jejunostomy (PEG-J) tube support, with the final esophagram confirming closure and restoration of swallowing function.

A subsequent esophagram at 16 weeks demonstrated complete closure without leakage, allowing safe removal of the PEG-J tube. By 20 weeks, outpatient follow-up revealed normal swallowing function and significant weight recovery, underscoring the successful non-surgical management of this esophageal perforation (Figure 10). 

## Discussion

This case highlights a successful multimodal endoscopic approach to managing an esophageal perforation and underscores the evolving role of endoscopy in a condition historically managed surgically. Advances in minimally invasive endoscopic therapies over recent decades have significantly improved patient outcomes, reducing overall mortality rates from approximately 30% historically to about 15% currently, largely due to the advent of fully covered stents, improved endoscopic techniques, and enhanced supportive care strategies [7,8]. Early intervention remains critical, with initiation of definitive treatment within 24 hours markedly improving survival; mortality rates decrease to around 7% when treated promptly compared to approximately 20% with delays beyond 24 hours [8]. Consequently, a non-surgical, endoscopic approach has increasingly become the preferred initial management strategy in hemodynamically stable patients, as it effectively seals perforations and minimizes morbidity associated with open surgical intervention [7,9].

FCSEMS plays a central role in managing esophageal perforations larger than 6 mm, offering clinical success rates consistently reported between 70% and 90% and significantly reducing extraluminal contamination by covering the defect while allowing oral nutrition during the healing phase [9]. Meta-analyses and recent multicenter studies further validate these high success rates and underscore the importance of timely stent placement to achieve optimal outcomes [9,10]. In this case, rapid deployment of a Merit EndoMAXX fully covered stent effectively contained mediastinal contamination and promoted primary healing. This stent was selected due to its distinct anti-migration design featuring external friction-enhancing struts and lower radial expansion force, specifically advantageous for stabilizing in the distal esophagus near the gastroesophageal junction, an area notably prone to migration [11,12]. Comparative studies indicate differences between EndoMAXX and the commonly used WallFlex stent; while WallFlex provides higher radial expansion beneficial in dilating strictures, EndoMAXX's design may reduce patient discomfort and pressure-induced injury, achieving lower migration rates in benign conditions with strategic placement [11,12].

Despite initial successful stenting, this patient subsequently developed a small persistent residual defect (~3 mm), underscoring the necessity of sequential therapeutic approaches. Such residual defects frequently occur and are optimally managed through adjunctive techniques like APC and mechanical clipping. The combination of APC, which ablates epithelialized fistula edges, and mechanical clipping, notably using robust devices such as the MANTIS clip, achieves exceptionally high closure rates (~98%) [13]. Literature highlights the effectiveness of this multimodal method, especially in cases where tissue edges are fibrotic or resistant to simple clip closure alone. The MANTIS clip’s enhanced anchoring strength significantly outperforms standard clips, ensuring secure and durable closure of residual leaks or fistulas [13].

The sequential, individualized endoscopic approach demonstrated here reflects broader consensus in the literature, consistently advocating for multiple-stage, tailored interventions rather than a single modality, as this strategy significantly reduces procedural morbidity and the potential need for surgical rescue. Although endoluminal vacuum-assisted therapy (EVT) was not employed in our patient, it represents a valuable adjunct or alternative, especially in cases with extensive mediastinal contamination or large, persistent leaks. EVT provides continuous negative pressure drainage, achieving closure success rates of 80% to 90%, superior to stenting alone in certain contexts, but requires repeated endoscopic procedures, thereby limiting its initial application primarily to complex or refractory cases [14,15]. Recent comparative analyses reinforce careful patient selection for EVT, recommending its use when simpler modalities are insufficient due to defect complexity or persistent infection [15].

A critical component of endoscopic management includes addressing stent migration, a common complication occurring in approximately 15% to 25% of FCSEMS placements due to the absence of a stricture to anchor the stent. Various anchoring techniques, including endoscopic sutures, OTSCs, or thread-anchored clips, significantly mitigate migration risks. In this case, employing a simple yet effective anchoring method prevented migration during the critical healing period. Furthermore, careful stent selection and opting for diameters between 20 and 23 mm have shown efficacy in maximizing tissue friction and reducing migration rates, particularly when combined with adjunctive anchoring [16,17].

Post-perforation esophageal strictures remain a significant concern following successful endoscopic management. Approximately 20% to 40% of patients treated endoscopically with fully covered stents develop symptomatic strictures, typically within four to 12 weeks after stent removal. These strictures often necessitate subsequent dilations, particularly in patients with extensive mucosal injury, persistent inflammation, or prolonged stent placement. Therefore, structured surveillance and prompt intervention within the initial three-month period post stenting are recommended to optimize swallowing outcomes and patient quality of life [18-20].

## Conclusions

This case exemplifies the efficacy and adaptability of contemporary multimodal endoscopic therapy in managing esophageal perforations. Strategic deployment of FCSEMS, followed by APC and advanced clipping techniques, effectively managed the perforation without the morbidity associated with traditional surgical intervention. The choice of endoscopic repair over surgery was guided by patient stability, minimal mediastinal contamination, and the reduced morbidity profile compared to surgery, which typically carries higher risks of complications and delayed functional recovery. This report highlights critical lessons, particularly the importance of sequential management and vigilance for residual defects post stenting. Continued advancements in endoscopic techniques and emerging tools like EVT and robust clipping devices promise further improvements in patient outcomes, reinforcing endoscopy as an increasingly preferred therapeutic strategy in appropriately selected patients.
